# Supplementary material for: A multifunctional GH39 glycoside hydrolase from the anaerobic gut fungus Orpinomyces sp. strain C1A
Source: PeerJ. 2016 Aug 3;4:e2289. doi: 10.7717/peerj.2289 (PMC4975031; doi:10.7717/peerj.2289)
Supplement: Supplemental Information 1 [file peerj-04-2289-s001.docx]

Supplementary document

**A multifunctional GH39 glycoside hydrolase from the anaerobic gut fungus *Orpinomyces* sp. strain C1A**

Authors: Jessica M. Morrison^1^, Mostafa S. Elshahed^1^, and Noha H. Youssef^1*^

**Table S1. Properties of enzymes with β-xylosidase activity.** Table is organized by origin, fungal, then bacterial, then plant. All values listed are from enzymatic activities against *p*-nitrophenyl-β-D-xylopyranoside, PNPX. For the “Known GH39-Family Enzyme?” column, a “+” indicates that the enzyme listed is known to be a member of the GH39-family, whereas a “-“ indicates that it either is not a GH39-family enzyme, or its classification is unknown. “-“, not reported in the study. Anaerobic fungi are highlighted.

| **Origin** | **Protein Name (Annotation)** | **Known GH39-family Enzyme?** | **Specific Activity**  **(U/mg)** | **Kinetics**  **Km**  **(mM)** | **Kinetics**  **Vmax**  **(U/mg)** | **Optimal pH** | **pH Stability** | **Optimal Temp. (°C)** | **Temp. Stability (°C)** | **Other Activities** | **Ref.** |
| --- | --- | --- | --- | --- | --- | --- | --- | --- | --- | --- | --- |
| *Orpinomyces* sp. strain C1A | Bgxg1 | **+** | 11.5 | 0.00485 | 127 | 6 | 4-12 | 39 | 4-70 | β-glucosidase  β-galactosidase  Xylanase | This  Study |
| *Acremonium persicinum* | β-glucosidase | - | 0.002 | - | - | 5.5 | - | - | - | β-glucosidase | (Pitson et al. 1997) |
| *Aspergillus awamori* | β-xylosidase | - | 34.1 | 0.003 | 476 | 6.5 | - | 70 | to 70 | Xylanase | (Kormelink et al. 1993) |
| *Aspergillus carnonarious* | β-xylosidase | - | 3.29 | 0.198 | 3.64 | 4 | 3.5-6.5 | 60 | to 50 | Arabinosidase | (Kiss & Kiss 2000) |
| *Aspergillus fumigatus* | β-xylosidase | - | 27.5 | 2 | - | 4.5 | 2-8 | 75 | to 65 | β-glucosidase | (Kitpreechavanich et al. 1986) |
|  | β-Glucosidase | - | 1.03 | - | - | 4.5 | 2-8 | 65 | to 60 | β-glucosidase | (Kitpreechavanich et al. 1986) |
| *Aspergillus niger* | β-xylosidase | - | 5.2 | 0.22 | - | 6.7-7 | - | 42 | to 46 | β-glucosidase | (John et al. 1979) |
|  | β-xylosidase 1 | - | 60.2 | - | - | - | - | - | - | β-glucosidase  Arabinosidase | (vanPeij et al. 1997) |
|  | β-xylosidase 2 | - | 60.9 | - | - | - | - | - | - | β-glucosidase  Arabinosidase | (vanPeij et al. 1997) |
| *Aspergillus nidulans* | β-xylosidase | - | 1.2 | 1.1 | 25.6 | 5 | 4-6 | 50 | to 45 | None | (Kumar & Ramon 1996) |
| *Aspergillus phoenicis* | β-xylosidase | - | 821 | 2.36 | - | 4-4.5 | 4-6 | 75 | to 60 | None | (Rizzatti et al. 2001) |
| *Aspergillus pulverulentus* | β-xylI | - | 32 | - | - | 2.5-3.5 | 1.5-6.5 | 60 | to 50 | None | (Sulistyo et al. 1995) |
|  | β-xylII | - | 12 | - | - | 4-5 | 1.5-6.5 | 60 | to 50 | None | (Sulistyo et al. 1995) |
| *Aureobasidium* sp. | β-xylosidase | - | 626 | 2 | 940 | 3.5 | 3.5-9 | 80 | to 80 | β-glucosidase | (Hayashi et al. 2001) |
| *Aureobasidium pullulans* | β-xylosidase | - | 7.3 | - | - | 4.5 | 2-9.5 | 80 | to 70 | None | (Dobberstein & Emeis 1991) |
| *Chaetomonium cellulolyticum* | Xylanase 3 | - | 0.03 | - | - | 6-7 | - | 50 | - | Xylanase  Arabinosidase  Endoglucanase | (Baraznenok et al. 1999) |
| *Fusarium oxysporum* | Xylanase | - | 0.5 | - | - | 7.4 | 5.8-8.2 | 50 | to 50 | Xylanase  Endoglucanase  Exoglucanase | (Christakopoulos et al. 1996) |
| *Fusarium proliferatum* | β-xylosidase | - | 53 | 0.77 | - | 4.5 | - | 60 | - | None | (Saha 2003) |
| *Fusarium vercillioides* | β-xylosidase | - | 57 | 0.85 | - | 4.5 | - | 65 | - | None | (Saha 2001) |
| *Humicola grisea* | β-glucosidase 1 | - | 4.38 | - | - | 4.5 | 4-12 | 60-65 | 30-60 | β-glucosidase  β-galactosidase  Cellobiohydrolase | (Takashima et al. 1996) |
|  | β-glucosidase 2 | - | 0.07 | - | - | 6-8 | 4-12 | 60-65 | 30-50 | β-glucosidase  β-galactosidase  Cellobiohydrolase | (Takashima et al. 1996) |
|  | β-glucosidase 3 | - | 0.08 | - | - | 6-8 | 4-12 | 60-65 | 30-50 | β-glucosidase  β-galactosidase  Cellobiohydrolase | (Takashima et al. 1996) |
|  | β-glucosidase 4 | - | 0.21 | - | - | 6-7 | 4-12 | 50-55 | 30-50 | β-glucosidase  β-galactosidase  Cellobiohydrolase | (Takashima et al. 1996) |
|  | β-glucosidase 5 | - | 0.36 | - | - | 6 | 4-12 | 60 | 30-55 | β-glucosidase  β-galactosidase  Cellobiohydrolase | (Takashima et al. 1996) |
|  | β-glucosidase 6 | - | 0.17 | - | - | 6-7 | 4-12 | 65-70 | 30-65 | β-glucosidase  β-galactosidase  Cellobiohydrolase | (Takashima et al. 1996) |
|  | β-xylosidase | - | 19.6 | 1.37 | 13 | 6.5 | 4-9 | 55 | to 50 | None | (Iembo et al. 2006) |
| *Neocallimastix frontalis* | β-xylosidase | - | 4.3 | 0.33 | - | 6.5 | - | 35 | - | Xylanase | (Hebraud & Fevre 1990) |
|  | β-xylosidase | - | 0.9 | 2.98 | 0.27 | 6.4 | - | 37 | to 40 | β-glucosidase  Arabinosidase | (Garcia-Campayo & Wood 1993) |
|  | β-xylosidase | - | 16 | 0.34 | - | 6.5 | 5-8 | 35 | to 40 | None | (Hebraud & Fevre 1988a) |
| *Neocallimastix patriciarum* | β-xylosidase I | - | 30.4 | 0.59 | 38 | 6 | 5-8 | 50 | 45-60 | Arabinosidase | (Zhu et al. 1994) |
|  | β-xylosidase II | - | 8.7 | 0.13 | 8.9 | 6 | 5-7 | 40 | 25-45 | None | (Zhu et al. 1994) |
| *Neurospora crassa* | β-xylosidase | - | 0.26 | 0.047 | - | 4.5-5 | - | 55 | - | Cellobiohydrolase | (Deshpande et al. 1986) |
| *Penicillium wortmanni* | Xylosidase 1 | - | 22 | 4.2 | 42.6 | 3-4.5 | - | 55-65 | - | None | (Matsuo et al. 1987) |
|  | Xylosidase 2 | - | 40 | 2.3 | 73.2 | 3-4.5 | - | 55-65 | - | None | (Matsuo et al. 1987) |
|  | Xylosidase 3 | - | 61.5 | 1.0 | 88.2 | 3-4.5 | - | 55-65 | - | None | (Matsuo et al. 1987) |
|  | Xylosidase 4 | - | 30.5 | 3.3 | 106.6 | 3-4.5 | - | 55-65 | - | None | (Matsuo et al. 1987) |
| *Pichia pastoris* X-33 | EXG1 | - | 11.6 | - | - | - | - | - | - | β-glucosidase  β-galactosidase | (Xu et al. 2006) |
| *Piromyces communis* | β-xylosidase | - | 28 | 0.4 | - | 6.0 | 5-8 | 39 | to 40 | None | (Hebraud & Fevre 1988a) |
| *Sphaeromonas communis* | β-xylosidase | - | 27 | 0.36 | - | 6.5 | 5-8 | 39 | to 40 | None | (Hebraud & Fevre 1988a) |
| *Talaomyces emersonii* | Xyl I | - | 3.0 | 0.13 | - | 2.5 | - | 60 | - | None | (Tuohy et al. 1993) |
|  | Xyl II | - | 92.9 | 32.9 | - | 4.2 | - | 78 | - | None | (Tuohy et al. 1993) |
|  | Xyl III | - | 0.2 | 1.4 | - | 3.5 | - | 67 | - | None | (Tuohy et al. 1993) |
| *Trichoderma harzianum* | β-xylosidase | - | 3.42 | 0.103 | - | 4-4.5 | - | 60 | - | β-glucosidase  β-galactosidase  Arabinosidase | (Ximenes et al. 1996) |
| *Trichoderma reesei* | CBHII | - | 0.00001 | - | - | - | - | - | - | β-glucosidase  Mannanase  Xylanase  Cellobiohydrolase  Exoglucanase | (Bailey et al. 1993) |
|  | EGI | - | 0.00001 | - | - | - | - | - | - | β-glucosidase  Mannanase  Xylanase  Cellobiohydrolase  Exoglucanase | (Bailey et al. 1993) |
|  | XYL 9 | - | 0.00002 | - | - | - | - | - | - | β-glucosidase  Mannanase  Xylanase | (Bailey et al. 1993) |
|  | XYL 5.5 | - | 0.00005 | - | - | - | - | - | - | β-glucosidase  Mannanase  Xylanase | (Bailey et al. 1993) |
|  | β-xylosidase | - | 470 | 0.08 | - | 4 | 3-7 | 60 | to 55 | Arabinosidase | (Poutanen & Puls 1988) |
| *Trichoderma viride* | β-xylosidase | - | 10.8 | - | - | 3.5 | 4-5 | 55 | to 65 | None | (Matsuo & Yasui 1984) |
| *Bacillus halodurans* C-125 | BH1068 | + | - | 30.1 | - | 6.5 | 5-7 | 47 | to 45 | None | (Wagschal et al. 2008) |
| *Bacillus stearothermophilus* | β-xylosidase | - | 34.2 | 0.0012 | - | 6 | 6-8 | 70 | to 60 | None | (Nanmori et al. 1990) |
| *Bacillus thermantarcticus* | β-xylosidase | - | 160 | 0.5 | - | 6.0 | - | 70 | to 60 | None | (Lama et al. 2004) |
| *Butyrivibrio fibrisolvens* | xylB | - | 8.9 | - | - | - | - | - | - | Arabinosidase | (Utt et al. 1991) |
| *Caulobacter crescentus* | CcXynB2 | **+** | 215 | 9.3 | 402 | 6 | 4.5-7.5 | 55 | to 50 | None | (Correa et al. 2012) |
| *Clostridium stercorarium* | β-glucosidase | - | 5.7 | - | - | 5.5 | 5-7 | 65 | to 75 | β-glucosidase  Cellobiohydrolase  Exoglucanase | (Bronnenmeier & Staudenbauer 1988) |
|  | xylA | - | 3.5 | 2.5 | 5.9 | 7 | 5-10 | 65 | - | Arabinosidase | (Sakka et al. 1993) |
| *Geobacillus stearothermophilus* | β-xylosidase | **+** | 133 | 2.38 | 147 | 6.5 | 5.5-7 | 70 | 50-70 | None | (Bhalla et al. 2014; Czjzek et al. 2005) |
| *Ruminococcus flavefaciens* | Exo A | - | 0.01 | - | - | 5 | 4.5-5.5 | - | - | Cellobiohydrolase  Exoglucanase | (Gardner et al. 1987) |
| *Thermoanaerobacterium*  *saccharolyticum* | β-xylosidase | **+** | 53.8 | 28 | 276 | 6 | 6-6.5 | 65 | to 67 | None | (Shao et al. 2011) |
| *Thermoanaerobacter* sp. | β-xylosidase | - | 66 | 0.038 | 183 | 5.9 | 5-8.5 | 93 | to 86 | Arabinosidase | (Shao & Wiegel 1992) |
| *Hordeum vulgare* | β-xylosidase | - | 6.06 | - | - | - | - | - | - | Arabinosidase | (Lee et al. 2003) |

**Table S2. Properties of enzymes with β-glucosidase activity.** Table is organized by origin, fungal, then bacterial, then plant. All values listed are from enzymatic activities against *p*-nitrophenyl-β-D-glucopyranoside, PNPG. “-“, not reported in the study. Rows of anaerobic fungi are highlighted.

| **Origin** | **Protein Name (Annotation)** | **Specific Activity**  **(U/mg)** | **Kinetics**  **Km**  **(mM)** | **Kinetics**  **Vmax**  **(U/mg)** | **Optimal pH** | **pH Stability** | **Optimal Temp. (°C)** | **Temp. Stability (°C)** | **Other Activities** | **Ref.** |
| --- | --- | --- | --- | --- | --- | --- | --- | --- | --- | --- |
| *Orpinomyces* sp. strain C1A | Bgxg1 | 73.4 | 0.000013 | 769 | 6 | 4-12 | 39 | 4-70 | β-xylosidase  β-galactosidase  Xylanase | This  Study |
| *Acremonium persicinum* | β-glucosidase | 0.0183 | 0.3 | - | 5.5 | - | - | - | Cellobiohydrolase | (Pitson et al. 1997) |
| *Aspergillus fumigatus* | β-xylosidase | 0.76 | - | - | 4.5 | 2-8 | 75 | to 65 | β-xylosidase | (Kitpreechavanich et al. 1986) |
|  | β-Glucosidase | 1.03 | - | - | 4.5 | 2-8 | 65 | to 60 | β-xylosidase | (Kitpreechavanich et al. 1986) |
| *Aspergillus nidulans* | B-Gluco-I | 4.52 | 0.24 | 8 | 5 | - | 65 | - | None | (Bagga et al. 1990) |
|  | B-Gluco-II | 0.55 | 0.12 | 0.7 | 6 | - | 35 | - | None | (Bagga et al. 1990) |
| *Aspergillus niger* | β-xylosidase | 0.15 | - | - | 6.7-7 | - | 42 | to 46 | β-xylosidase | (John et al. 1979) |
|  | β-xylosidase 1 | 0.2 | - | - | - | - | - | - | β-xylosidase  Arabinosidase | (vanPeij et al. 1997) |
|  | β-xylosidase 2 | 0.3 | - | - | - | - | - | - | β-xylosidase  Arabinosidase | (vanPeij et al. 1997) |
| *Aureobasidium* sp. | β-xylosidase | 15.6 | - | - | 3.5 | 3.5-9 | 80 | to 80 | β-xylosidase | (Hayashi et al. 2001) |
| *Candida peltata* | β-glucosidase | 108 | 2.3 | 221 | 5 | 3.5-6 | 50 | 25-45 | Cellobiohydrolase | (Saha & Bothast 1996) |
| *Chrysosporium lucknowense* | EG60 | 0.12 | - | - | 4.5-6 | 5-7 | 60-70 | 60-75 | Endoglucanase | (Bukhtojarov et al. 2004) |
|  | CBH1 | 0.02 | - | - | 4.5-6 | 5-7 | 60-70 | 60-75 | Cellobiohydrolase | (Bukhtojarov et al. 2004) |
| *Fusarium oxysporum* | Xylanase | 0.9 | - | - | 7.4 | 5.8-8.2 | 50 | to 50 | Xylanase  Endoglucanase  Exoglucanase | (Christakopoulos et al. 1996) |
| *Fusarium solani* | Cellulase | 4.65 | - | - | 5 | 5.7 | 65 | to 65 | Cellobiohydrolase  Endoglucanase  Exoglucanase | (Wood 1971) |
| *Humicola grisea* | Exoglucanase 1 | 1.14 | - | - | 6-8 | 4-14 | 55-70 | 30-75 | Cellobiohydrolase  Endoglucanase  Exoglucanase | (Takashima et al. 1996) |
|  | β-glucosidase 1 | 85.7 | 0.16 | 64 | 4.5 | 4-12 | 60-65 | 30-60 | Cellobiohydrolase  β-galactosidase  β-xylosidase | (Takashima et al. 1996) |
|  | β-glucosidase 2 | 12.5 | 0.25 | 21 | 6-8 | 4-12 | 60-65 | 30-50 | Cellobiohydrolase  β-galactosidase  β-xylosidase | (Takashima et al. 1996) |
|  | β-glucosidase 3 | 12.6 | 0.22 | 23 | 6-8 | 4-12 | 60-65 | 30-50 | Cellobiohydrolase  β-galactosidase  β-xylosidase | (Takashima et al. 1996) |
|  | β-glucosidase 4 | 8.34 | 0.34 | 8.7 | 6-7 | 4-12 | 50-55 | 30-50 | Cellobiohydrolase  β-galactosidase  β-xylosidase | (Takashima et al. 1996) |
|  | β-glucosidase 5 | 17.9 | 0.56 | 18 | 6 | 4-12 | 60 | 30-55 | Cellobiohydrolase  β-galactosidase  β-xylosidase | (Takashima et al. 1996) |
|  | β-glucosidase 6 | 33.4 | 0.12 | 40 | 6-7 | 4-12 | 65-70 | 30-65 | Cellobiohydrolase  β-galactosidase  β-xylosidase | (Takashima et al. 1996) |
|  | BGL I | 26.1 | 0.34 | 25 | 6 | 6-11 | 55 | to 50 | Cellobiohydrolase  β-galactosidase | (Takashima et al. 1999) |
| *Neocallimastix frontalis* | β-xylosidase | 0.2 | 2.98 | 0.27 | 6.4 | - | 37 | to 40 | β-xylosidase  Arabinosidase | (Garcia-Campayo & Wood 1993) |
|  | β-glucosidase | 9.0 | 0.031 | - | - | - | - | - | β-galactosidase  Cellobiohydrolas | (Wilson et al. 1994) |
|  | β-glucosidase | 15 | 0.55 | - | 6 | 5-8 | 50 | to 40 | None | (Hebraud & Fevre 1988a) |
| *Orpinomyces* sp. strain PC-2 | β-glucosidase | 33.5 | 0.39 | 47.5 | 6.2 | 5-8 | 50 | to 65 | Cellobiohydrolase | (Chen et al. 1994) |
| *Paecilomyces thermophila* | β-glucosidase | 97.2 | 0.27 | 780 | 6.2 | 5-8.5 | 75 | 65-70 | Exoglucanase | (Yang et al. 2008) |
| *Penicillium funiculosum* | Cellulase | - | 0.77 | - | 5 | - | 50 | to 60 | Cellobiohydrolase  Exoglucanase | (Wood & Mccrae 1982a) |
| *Phanerochaete chrysosporium* | β-glucosidase | - | 0.096 | - | 4-5.2 | 4-8 | 25 | to 45 | None | (Lymar et al. 1995) |
| *Pichia pastoris* X-33 | EXG1 | 37.5 | - | - | - | - | - | - | β-xylosidase  Exoglucanase | (Xu et al. 2006) |
| *Piromyces communis* | β-glucosidase | 20 | 0.52 | - | 6 | 5-8 | 50 | to 40 | None | (Hebraud & Fevre 1988a) |
| *Piromyces* sp. E2 | β-glucosidase | 0.25 | 1.0 | 66.5 | 5.7-6.3 | - | 47 | to 37 | Cellobiohydrolase | (Harhangi et al. 2002) |
| *Sphaeromonas communis* | β-glucosidase | 46 | 0.51 | - | 6 | 5-8 | 50 | to 40 | None | (Hebraud & Fevre 1988a) |
| *Sporotrichum thermophila* | β-glucosidase I | 89 | 0.29 | - | 5.4 | 4-6.5 | 65 | to 65 | None | (Bhat et al. 1993) |
|  | β-glucosidase A | 0.595 | 0.5 | - | 5.6 | - | 50 | to 40 | None | (Meyer & Canevascini 1981) |
|  | β-glucosidase B | 1.295 | 0.18 | - | 6.3 | - | 50 | to 40 | None | (Meyer & Canevascini 1981) |
| *Stachybotrys* sp. | β-glucosidase p21 | 0.33 | 0.27 | 78 | 5 | 5-7 | 50 | to 60 | None | (Amouri & Gargouri 2006) |
| *Thermoascus aurantiacus* | β-glucosidase | 232 | 0.52 | 6500 | 5 | 6-8 | 70 | to 75 | None | (Tong et al. 1980) |
|  | β-glucosidase | 0.3 | 0.11 | - | 4.5 | 4.4-5.2 | 80 | 70-90 | Cellobiohydrolase | (Parry et al. 2001) |
| *Trichoderma harzianum* | BGL | 0.35 | - | - | - | - | 28 | - | None | (Ahmed et al. 2009) |
|  | β-xylosidase | 0.28 | - | - | 4-4.5 | - | 60 | - | β-xylosidase  β-galactosidase  Arabinosidase | (Ximenes et al. 1996) |
| *Trichoderma koningii* | β-glucosidase | 16 | 0.37 | - | 4-5 | 4-6 | 45 | to 65 | Cellobiohydrolase | (Wood & Mccrae 1982b) |
|  | β-glucosidase II | 14 | 0.85 | - | 4-5 | 4-6 | 45 | to 65 | Cellobiohydrolase | (Wood & Mccrae 1982b) |
| *Trichoderma reesei* | BGL2 | 23.9 | 2.22 | 40 | 6 | 6-11 | 45 | to 45 | Cellobiohydrolase  β-galactosidase | (Takashima et al. 1999) |
|  | BGLI | 767.7 | - | - | 4 | 5-9 | 70 | to 55 | Cellobiohydrolase | (Takashima et al. 1998) |
|  | CBH I | 5.5 | - | - | - | - | - | - | Cellobiohydrolase  Endoglucanase | (Woodward et al. 1994) |
|  | CBH II | 33 | - | - | - | - | - | - | Cellobiohydrolase  Endoglucanase | (Woodward et al. 1994) |
|  | CBH III | 0.00001 | - | - | - | - | - | - | Cellobiohydrolase  Xylanase  Mannanase  β-xylosidase  Exoglucanase | (Bailey et al. 1993) |
|  | EG I | 0.00005 | - | - | - | - | - | - | Cellobiohydrolase  Xylanase  Mannanase  β-xylosidase  Exoglucanase | (Bailey et al. 1993) |
|  | XYL 9 | 0.00001 | - | - | - | - | - | - | Xylanase  Mannanase  β-xylosidase | (Bailey et al. 1993) |
|  | XYL 5.5 | 0.000001 | - | - | - | - | - | - | Xylanase  Mannanase  β-xylosidase | (Bailey et al. 1993) |
| *Trichoderma viride* T100-14 | β-glucosidase | 5.6 | - | - | - | - | - | - | Cellobiohydrolase | (Zhou et al. 2008) |
| *Bacillus* sp. KSM-S237 | Cellulase | 5.4 | - | - | 8.6-9 | 5-11 | 45 | to 60 | Endoglucanase  Cellobiohydrolase  Exoglucanase  Xylanase | (Hakamada et al. 1997) |
| *Bacillus pumilus* EB3 | Cellulase | 0.038 | - | - | 6 | 5-9 | 60 | 30-70 | Exoglucanase  Endoglucanase | (Ariffin et al. 2006) |
| *Bacillus sphaericus* JS1 | Cellulase | 3.07 | - | - | 8 | 7-10.5 | 60 | to 70 | Endoglucanase | (Singh et al. 2004) |
| *Cellulomonas fimi* | CenA | 1.6 | - | - | - | - | - | - | Exoglucanase  Endoglucanase  Cellobiohydrolase | (Tomme et al. 1996) |
|  | Exg | 0.9 | - | - | 6 | 5-9 | - | 60-75 | Xylanase  Endoglucanase  Exoglucanase  Cellobiohydrolase | (Curry et al. 1988) |
| *Clostridium stercorarium* | β-glucosidase | 31.6 | 0.8 | - | 5.5 | 5-7 | 65 | to 75 | Cellobiohydrolase | (Bronnenmeier & Staudenbauer 1988) |
| *Clostridium thermocellum* | β-glucosidase | 127 | - | - | 6-6.5 | 5.5-7.5 | 60 | to 68.5 | Cellobiohydrolase | (Ait et al. 1982) |
| *Thermotoga neapolitana* | CelA | 0.01 | - | - | 6 | - | 95 | to 95 | Endoglucanase  Cellobiohydrolase | (Bok et al. 1998) |
|  | CelB | 0.18 | - | - | 6-6.6 | - | 106 | to 106 | Endoglucanase  Cellobiohydrolase | (Bok et al. 1998) |
| *Panesthia cribrata* | GD1 | 9 | 10.6 | - | - | - | - | - | Endoglucanase | (Scrivener & Slaytor 1994) |
|  | GD2 | 3 | 13.8 | - | - | - | - | - | Endoglucanase | (Scrivener & Slaytor 1994) |

**Table S3. Properties of enzymes with β-galactosidase activity.** Table is organized by origin, fungal, then bacterial, then plant. All values listed are from enzymatic activities against *p*-nitrophenyl-β-D-galactopyranoside, PNPGal. “-“, not reported in the study. Rows of anaerobic fungi are highlighted.

| **Origin** | **Protein Name (Annotation)** | **Specific Activity**  **(U/mg)** | **Kinetics**  **Km**  **(mM)** | **Kinetics**  **Vmax**  **(U/mg)** | **Optimal pH** | **pH Stability** | **Optimal Temp. (°C)** | **Temp. Stability (°C)** | **Other Activities** | **Ref.** |  |
| --- | --- | --- | --- | --- | --- | --- | --- | --- | --- | --- | --- |
| *Orpinomyces* sp. strain C1A | Bgxg1 | 54.6 | 0.000214 | 769 | 6 | 4-12 | 39 | 4-70 | β-glucosidase  β-xylosidase  Xylanase | This  Study |  |
| *Aspergillus aculeatus* | β-galactosidase | 24 | 3.28 | 284 | 5.4 | - | 55-60 | - | None | (van Casteren et al. 2000) |  |
| *Aspergillus niger* | β-galactosidase | 33.3 | 1.3 | 51.2 | 4 | 3.5-4.5 | 60-65 | to 50 | None | (Manzanares et al. 1998) |  |
| *Humicola grisea* | β-glucosidase 1 | 0.15 | - | - | 4.5 | 4-12 | 60-65 | 30-60 | Cellobiohydrolase  β-glucosidase  β-xylosidase | (Takashima et al. 1996) |  |
|  | β-glucosidase 2 | 0.03 | - | - | 6-8 | 4-12 | 60-65 | 30-50 | Cellobiohydrolase  β-glucosidase  β-xylosidase | (Takashima et al. 1996) |  |
|  | β-glucosidase 3 | 0.04 | - | - | 6-8 | 4-12 | 60-65 | 30-50 | Cellobiohydrolase  β-glucosidase  β-xylosidase | (Takashima et al. 1996) |  |
|  | β-glucosidase 4 | 2.70 | - | - | 6-7 | 4-12 | 50-55 | 30-50 | Cellobiohydrolase  β-glucosidase  β-xylosidase | (Takashima et al. 1996) |  |
|  | β-glucosidase 5 | 0.03 | - | - | 6 | 4-12 | 60 | 30-55 | Cellobiohydrolase  β-glucosidase  β-xylosidase | (Takashima et al. 1996) |  |
|  | β-glucosidase 6 | 0.08 | - | - | 6-7 | 4-12 | 65-70 | 30-65 | Cellobiohydrolase  β-glucosidase  β-xylosidase | (Takashima et al. 1996) |  |
|  | BGL1 | 11.2 | 1.82 | 21.1 | 6 | 6-11 | 55 | to 50 | Cellobiohydrolase  β-glucosidase | (Takashima et al. 1999) |  |
| *Hypocrea jecorina* | β-galactosidase | 0.828 | 0.36 | 144.6 | 5 | 3-6 | 60 | to 65 | None | (Gamauf et al. 2007) |  |
| *Kluyveromyces fragilis* | β-galactosidase | 2.5 | - | - | 6.5-6.8 | 6-7 | 40 | to 40 | None | (Ladero et al. 2002) |  |
| *Neocallimastix frontalis* | β-glucosidase | 0.1 | - | - | - | - | - | - | β-glucosidase  Cellobiohydrolase | (Wilson et al. 1994) |  |
|  | β-galactosidase | 3 | 1.89 | - | 6 | 5-7 | 50 | to 40 | None | (Hebraud & Fevre 1988b) |  |
| *Pichia pastoris* X-33 | EXG1 | 0.232 | - | - | - | - | - | - | β-glucosidase  β-xylosidase | (Xu et al. 2006) |  |
| *Piromyces communis* | β-galactosidase | 2 | 1.24 | - | 6 | 5-7 | 50 | to 40 | None | (Hebraud & Fevre 1988b) |  |
| *Sphaeromonas communis* | β-galactosidase | 3.7 | 1.2 | - | 6 | 5-7 | 50 | to 40 | None | (Hebraud & Fevre 1988b) |  |
| *Sterigmatomyces elviae* | β-galactosidase | 20.2 | 9.5 | 96 | 4.5-5 | 2.5-7 | 85 | to 80 | None | (Onishi & Tanaka 1995) |  |
| *Thermus* sp. | β-galactosidase | 5.4 | - | - | 5 | 5-11 | 90 | to 80 | None | (Ladero et al. 2002) |  |
| *Trichoderma harzianum* | β-xylosidase | 1.54 | - | - | 4-4.5 | - | 60 | - | β-xylosidase  β-glucosidase  Arabinosidase | (Ximenes et al. 1996) |  |
| *Trichoderma reesei* | BGL2 | 1.72 | 20 | 20.8 | 6 | 6-11 | 45 | to 45 | Cellobiohydrolase  β-glucosidase | (Takashima et al. 1999) |  |
| *Alicyclobacillus acidocaldarius* | β-galactosidase | 229 | 6 | - | 5.8 | 5-10.5 | 70 | to 70 | None | (Yuan et al. 2008) |  |
| *Bifidobacterium adolescentis* | β-galactosidase | 526 | 60 | 1129 | 6 | - | 50 | to 40 | None | (Hinz et al. 2004) |  |
| *Streptococcosu lactis* | β-galactosidase | 0.75 | - | - | - | - | - | - | None | (Citti et al. 1965) |  |
| *Thermus aquaticus* | β-galactosidase | 1750 | 0.002 | - | 5 | - | 80 | to 70 | None | (Ulrich et al. 1972) |  |

**Table S4. Properties of enzymes with xylanase activity.** Table is organized by origin, fungal, then bacterial, then plant. All values listed are from enzymatic activities against xylan. “-“, not reported in the study. Rows of anaerobic fungi are highlighted.

| **Origin** | **Protein Name (Annotation)** | **Specific Activity**  **(U/mg)** | **Kinetics**  **Km**  **(mg/mL)** | **Kinetics**  **Vmax**  **(U/mg)** | **Optimal pH** | **pH Stability** | **Optimal Temp. (°C)** | **Temp. Stability (°C)** | **Other Activities** | **Ref.** |  |
| --- | --- | --- | --- | --- | --- | --- | --- | --- | --- | --- | --- |
| *Orpinomyces* sp. strain C1A | Bgxg1 | 10.8 | 0.038 | 25.6 | 6 | 4-12 | 39 | 4-70 | β-glucosidase  β-xylosidase  β-galactosidase | This  Study |  |
| *Acrophialophora nainiana* | Xyn III | 31.25 | 4.37 | 0.24 | 6.5 | - | 50 | to 55 | None | (Cardoso & Ferreira 2003) |  |
|  | Xyn II | 33.2 | 40.9 | - | 7 | - | 55 | to 55 | None | (Salles et al. 2000) |  |
|  | Xyn I | 13.6 | 0.731 | - | 6 | - | 50-60 | to 50 | None | (Ximenes et al. 1999) |  |
| *Aspergillus awamori* | β-xylosidase | 1.7 | - | - | 6.5 | - | 70 | to 70 | β-xylosidase | (Kormelink et al. 1993) |  |
| *Aspergillus niger* | Xylanase I | 18.9 | - | - | 6-6.5 | - | 65-80 | to 80 | Exoglucanase | (John et al. 1979) |  |
|  | Xylanase IA | 35.2 | - | - | 5.5-6 | - | 65-80 | to 80 | Exoglucanase | (John et al. 1979) |  |
|  | Xylanase II | 24.5 | - | - | 4-4.5 | - | 65-80 | to 80 | None | (John et al. 1979) |  |
|  | Xylanase IIA | 48.0 | - | - | 4 | - | 65-80 | to 80 | None | (John et al. 1979) |  |
| *Chaetomonium cellulolyticum* | Xylanase 1 | 22 | - | - | 6-7 | - | 50 | - | Endoglucanase  Exoglucanase | (Baraznenok et al. 1999) |  |
|  | Xylanase 2 | 18 | - | - | 6-7 | - | 50 | - | Endoglucanase  Exoglucanase | (Baraznenok et al. 1999) |  |
|  | Xylanase 3 | 11 | - | - | 6-7 | - | 50 | - | Endoglucanase  Arabinosidase | (Baraznenok et al. 1999) |  |
| *Chrysosporium lucknowense* | EG28 | 0.2 | - | - | 4.5-6 | 5-7 | 60-70 | 60-75 | Endoglucanase  Exoglucanase | (Bukhtojarov et al. 2004) |  |
|  | EG44 | 0.07 | - | - | 4.5-6 | 5-7 | 60-70 | 60-75 | Endoglucanase  Exoglucanase | (Bukhtojarov et al. 2004) |  |
|  | EG47 | 0.08 | - | - | 4.5-6 | 5-8.5 | 60-70 | 60-75 | Endoglucanase  Exoglucanase | (Bukhtojarov et al. 2004) |  |
|  | EG51 | 0.18 | - | - | 4.5-6 | 5-7 | 60-70 | 60-75 | Endoglucanase  Exoglucanase | (Bukhtojarov et al. 2004) |  |
|  | CBHII | 1.4 | - | - | 4.5-6 | 5-7 | 60-70 | 60-75 | Cellobiohydrolase  Exoglucanase | (Bukhtojarov et al. 2004) |  |
| *Fusarium oxysporum* | Xylanase | 5.2 | 3.8 | - | 7.4 | 5.8-8.2 | 50 | to 50 | Endoglucanase  Exoglucanase | (Christakopoulos et al. 1996) |  |
| *Gloephyllum trabeum* | Cel5A | 0.77 | - | - | - | - | - | - | Endoglucanase  Exoglucanase | (Cohen et al. 2005) |  |
|  | Xyn10A | 390 | - | - | - | - | - | - | Endoglucanase  Exoglucanase | (Cohen et al. 2005) |  |
|  | Cel12A | 0.88 | - | - | - | - | - | - | Endoglucanase  Exoglucanase | (Cohen et al. 2005) |  |
| *Humicola grisea* | X1 | - | 7.233 |  | 5 |  | 50 | to 50 | None | (Lucena-Neto & Ferreira 2004) |  |
|  | X2 | 10.23 | 10.87 | - | 4.5-6.5 | - | 55-60 | to 60 | None | (Lucena-Neto & Ferreira 2004) |  |
|  | Xylanase | 169.4 | 3.3 | 229 | 5.5 | - | 70 | to 50 | None | (Monti et al. 1991) |  |
| *Neocallimastix frontalis* | Xylanase | 0.36 | 1.13 | - | 6 | 3-8 | 50 | to 40 | None | (Hebraud & Fevre 1988b) |  |
| *Orpinomyces joyonii* | Cellulase | 5.2 | - | - | 5.5 | 5-7.5 | 40 | to 50 | Exoglucanase  Endoglucanase  Cellobiohydrolase | (Ye et al. 2001) |  |
| *Paecilomyces thermophila* | Xylanase | 2063 | 2 | 2344 | 7 | 6-10 | 75-80 | to 75 | None | (Li et al. 2006) |  |
| *Penicillium brasilianum* | XYL | 279 | - | - | - | - | - | - | None | (Jorgensen et al. 2003) |  |
| *Penicillium citrinum* | Xylanase | 360.7 | - | - | 8.5 | 4-10 | 50 | 0-60 | None | (Dutta et al. 2007) |  |
| *Piromyces communis* | Xylanase | 0.37 | 1 | - | 5.5 | - | 50 | - | None | (Hebraud & Fevre 1988b) |  |
| *Sphaeromonas communis* | Xylanase | 0.43 | 0.98 | - | 6 | - | 50 | - | None | (Hebraud & Fevre 1988b) |  |
| *Trichoderma harzianum* | XYL2 | - | 13.66 | - | 5 | 3.5-6.5 | 45 | to 50 | None | (Silveira et al. 1999) |  |
|  | Xylanase | 2400 | - | - | - | - | - | - | None | (Tan et al. 1987) |  |
| *Trichoderma reesei* | EG I | 0.55 | - | - | - | - | - | - | Endoglucanase  Cellobiohydrolase  β-glucosidase | (Suurnakki et al. 2000) |  |
|  | EG II | 0.003 | - | - | - | - | - | - | Endoglucanase  Cellobiohydrolase  β-glucosidase | (Suurnakki et al. 2000) |  |
|  | CBH I | 0.0003 | - | - | - | - | - | - | Endoglucanase  Cellobiohydrolase  β-glucosidase | (Suurnakki et al. 2000) |  |
|  | CBH II | 0.002 | - | - | - | - | - | - | Endoglucanase  Cellobiohydrolase  β-glucosidase | (Suurnakki et al. 2000) |  |
|  | CBH III | 0.0002 | - | - | - | - | - | - | Endoglucanase  β-glucosidase  β-xylosidase | (Bailey et al. 1993) |  |
|  | EG I | 0.29 | - | - | - | - | - | - | Endoglucanase  β-glucosidase  β-xylosidase | (Bailey et al. 1993) |  |
|  | XYL 9 | 11.33 | - | - | - | - | - | - | Endoglucanase  β-glucosidase  β-xylosidase | (Bailey et al. 1993) |  |
|  | XYL 5.5 | 3.855 | - | - | - | - | - | - | Endoglucanase  β-glucosidase  β-xylosidase | (Bailey et al. 1993) |  |
|  | EG I | 46.2 | - | - | 4 | 2-8 | 60 | to 45 | Endoglucanase  Exoglucanase  Cellobiohydrolase | (Takashima et al. 1998) |  |
| *Anaerocellum thermophilum* | CelA | 0.372 | - | - | 5-6 | - | 85-95 | - | Endoglucanase  Exoglucanase | (Zverlov et al. 1998) |  |
| *Bacillus amyoliquefaciens* | Cellulase | 22.5 | - | - | 7 | 4-9 | 50 | 40-80 | Endoglucanase  Exoglucanase  Cellobiohydrolase  β-glucosidase | (Lee et al. 2008) |  |
| *Bacillus circulans* | XylA | 2039.5 | 4 | 2667 | 6-6.5 | 4-8 | 75-80 | to 65 | None | (Dhillon et al. 2000) |  |
|  | XylB | 6423 | 25 | 200000 | 6-6.5 | 4-8 | 65-70 | to 65 | None | (Dhillon et al. 2000) |  |
| *Bacillus licheniformis* | Xylanase | 28.7 | 3.33 | 111 | 6-7.5 | 5-8 | 60 | to 60 | None | (Archana & Satyanarayana 2003) |  |
| *Bacillus stearothermophilus* | Xylanase | 122 | 3.8 | - | 7 | 5-11 | 60 | to 60 | None | (Nanmori et al. 1990) |  |
| *Bacillus subtilis* | Cellulase | 980 | - | - | 6 | 6-7.5 | 50-60 | to 50 | Endoglucanase | (Yin et al. 2010) |  |
| *Bacillus thermantarcticus* | Xylanase | 141 | 1.6 | - | 5.6 | - | 80 | to 60 | None | (Lama et al. 2004) |  |
| *Caldibacillus cellulovorans* | CMCase | 4.56 | - | - | 6.5-7 | - | 80 | to 70 | Endoglucanase  Exoglucanase | (Huang & Monk 2004) |  |
| *Cellulomonas fimi* | Exg | 37 | - | - | 6 | 5-9 | - | 60-75 | Endoglucanase  Cellobiohydrolase | (Curry et al. 1988) |  |
| *Clostridium stercorarium* | Avicelase II | 0.340 | - | - | 5-6 | 4.5-7 | 75 | 65-80 | Exoglucanase | (Bronnenmeier et al. 1991) |  |
| *Streptomyces lividans* | Xylanase | 364 | 0.78 | 850 | 6 | 5.5-7 | 60 | 0-37 | None | (Morosoli et al. 1986) |  |
| *Streptomyces rameus* | Xylanase | 4326 | 5.8 | - | 5.3 | 4.3-6.7 | 70 | 40-65 | None | (Li et al. 2010) |  |
| *Streptomyces thermoviolaceus* | STX-I | 1460 | - | - | 7 | 5-9 | 70 | to 50 | None | (Tsujibo et al. 1992) |  |
|  | STX-II | 1405 | - | - | 7 | 5-9 | 60 | to 60 | None | (Tsujibo et al. 1992) |  |
| *Thermatoga maritima* | CelA | 2.5 | - | - | 5 | 4-9 | 85-90 | to 95 | Endoglucanase  Exoglucanase  Cellobiohydrolase | (Liebl et al. 1996) |  |
|  | CelB | 0.008 | - | - | 7 | 5-9 | 85-90 | to 95 | Endoglucanase  Exoglucanase  Cellobiohydrolase | (Liebl et al. 1996) |  |
| *Thermatoga neapolitana* | CelB | 37 | - | - | 6-6.6 | - | 106 | to 106 | Endoglucanase  Exoglucanase  Cellobiohydrolase | (Bok et al. 1998) |  |

**Supplementary figures.**

**
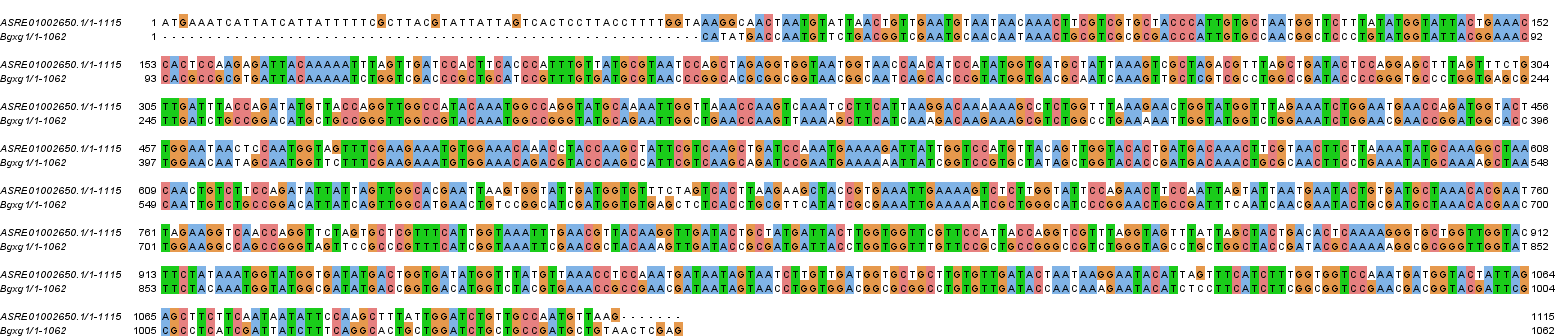
**

**Figure S1. Alignment of native Bgxg1 from *Orpinomyces* sp. strain C1A (ASRE01002650.1, range: 2346-3460) and codon-optimized Bgxg1 (for expression in *E. coli*).**

**
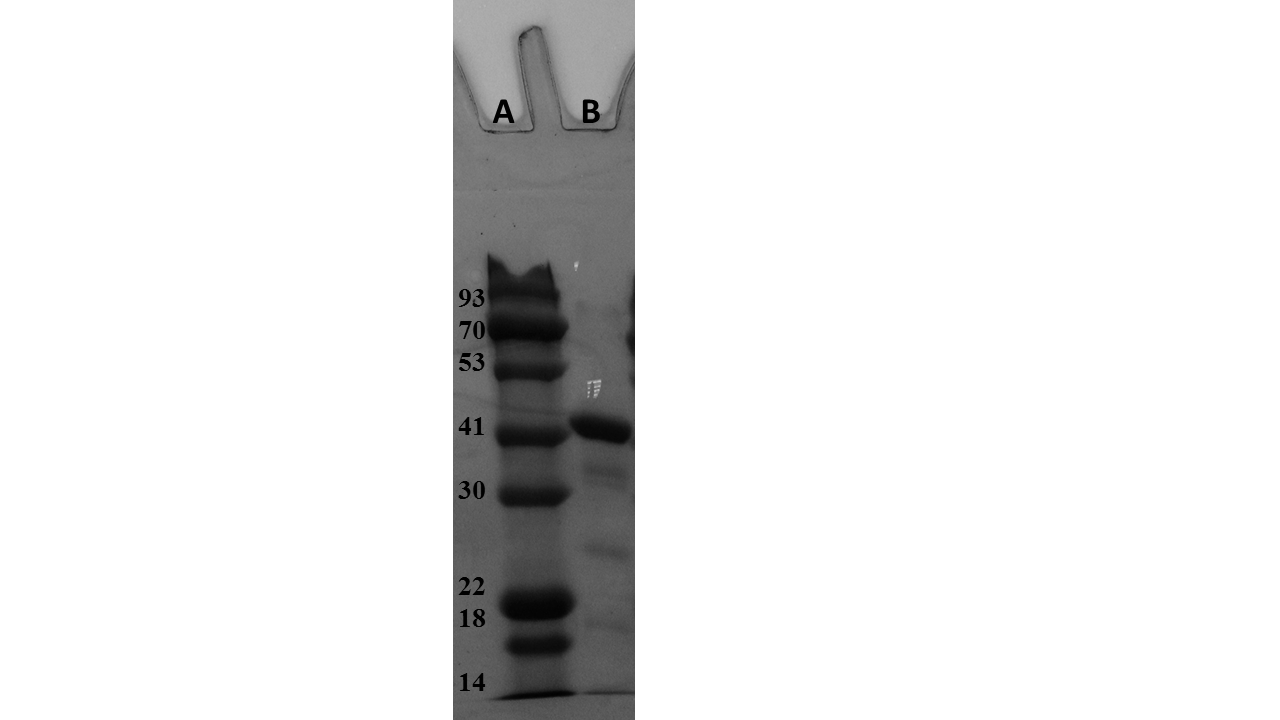
**

**Figure S2. SDS-PAGE analysis for Inclusion Body.** A 12.5% SDS-PAGE analysis of Inclusion Body Fraction stained with Coomassie blue. Lane A, Pre-stained Protein Ladder (Caisson Labs, Smithfield, Utah). Lane B, Inclusion Body Fraction. Note: Bgxg1 is 42.7 kDa.

**
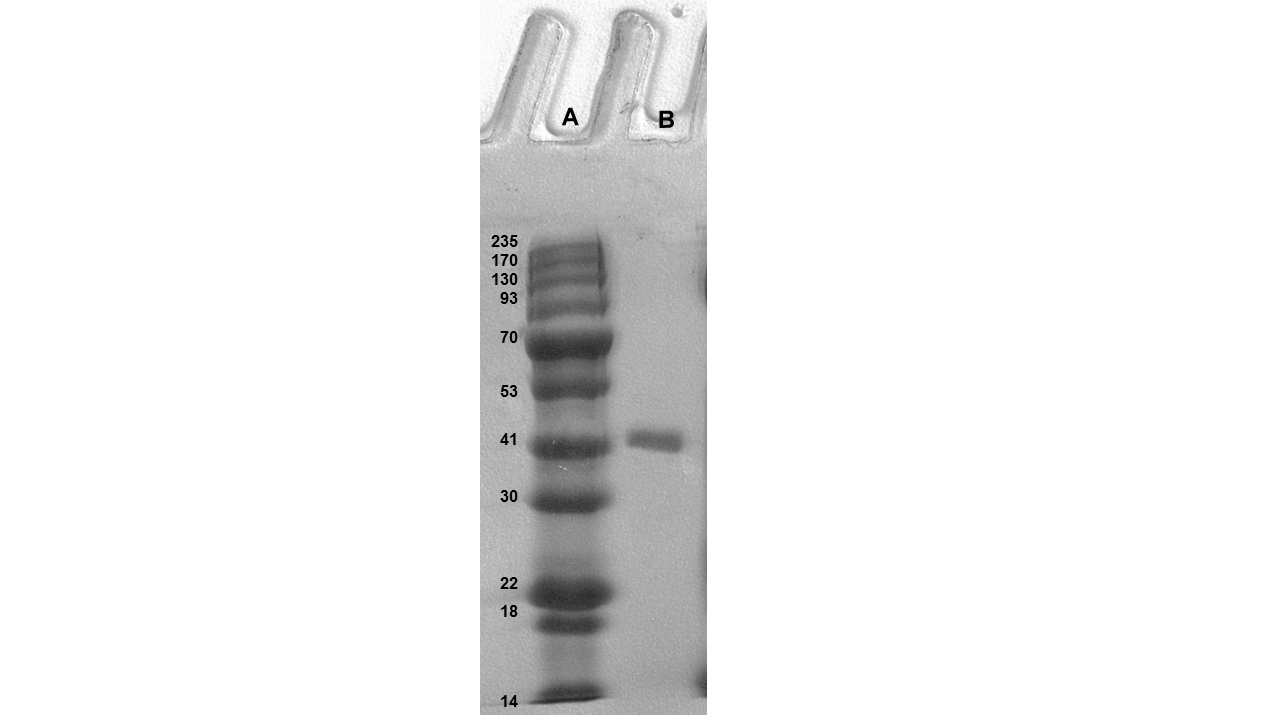
**

**Figure S3. SDS-PAGE analysis of Bgxg1.** A 12.5% SDS-PAGE analysis of recombinant Bgxg1 protein stained with Coomassie blue. Lane A, Pre-stained Protein Ladder (Caisson Labs, Smithfield, Utah). Lane B, Purified Bgxg1.

**Figure S4. Bgxg1 model and comparison of residues of interest. A**. Bgxg1 model obtained using I-TASSER. Residues of the α-helical domain are in blue; residues of the (α/β)_8_ barrel are in green; residues of the β-sandwich are in red. Active site residues (Asn126-Glu127-Pro128, Glu225) are shown in orange. **B-S** Changes in the area surrounding the active site between Bgxg1 (top panel) and 1UHV of *Thermoanaerobacter saccharolyticum* (bottom panel). **B and C**, Val46 (Bgxg1) vs Tyr50 (1UHV). **D and E,** Asp129 (Bgxg1) vs Asn162 (1UHV). **F and G,** Thr131 (Bgxg1) vs Phe166 (1UHV). **H and I,** Phe139 (Bgxg1) vs Tyr175 (1UHV). **J** **and K,** Cys163 (Bgxg1) vs Ala199 (1UHV). **L and M,** Lys171 (Bgxg1) vs Trp207 (1UHV). **N** **and O,** Leu194 (Bgxg1) vs Tyr230 (1UHV). **P and Q,** Arg242 (Bgxg1) vs Ala296 (1UHV). **S** Glu322 and Glu323 of 1UHV align with a gap in Bgxg1, but Gly266-Arg267 are predicted to occupy approximately the same position in Bgxg1 **R**. Modeling clearly suggests that these changes could putatively impact the size, charge, and/or polarity within the active site. Changes in polarity in and around the active site of a protein can directly affect the affinity of the protein for the substrate (Macgregor & Weber 1986). Changes that affect polarity include polar Tyr (1UHV, panel I) to nonpolar Phe139 (Bgxg1, panel H), as well as nonpolar Ala (1UHV, panel K) to polar, thiol-containing Cys163 (Bgxg1, panel J). Changes in charge in and around the active site can change substrate binding and intermediate stabilization (Czjzek et al. 2005). Changes that affect charge include neutral Asn (1UHV, panel E) to negative Asp129 (Bgxg1, panel D). Changes in the size of the active site can also have a direct impact on substrate binding. Combinations of the three aforementioned changes were seen. Changes in both size and polarity include: large, polar Tyr (1UHV, panel C) to small, nonpolar Val46 (Bgxg1, panel B); large, nonpolar Phe (1UHV, panel G) to small, polar Thr131 (Bgxg1, panel F); large, polar Tyr (1UHV, panel O) to small, nonpolar Leu194 (Bgxg1, panel N). A change in both charge and polarity includes nonpolar, neutral Trp (1UHV, panel M) to polar, positively Lys171 (Bgxg1, panel L). Finally, a change in charge, size, and polarity includes neutral, small, nonpolar Ala (1UHV, panel Q) to positively charged, large, and polar Arg242 (Bgxg1, panel P). Lastly, the final two changes represent deletions/gaps in the Bgxg1 sequence as opposed to negatively charged glutamic acids in the other four sequences (Table 4, panel S). However, structural modeling suggests that in lieu of the Glu322-Glu323 residues in 1UHV, Bgxg1 has Gly266 and Arg267 (panel R). This represents a significant change in charge, from negatively charged Glu to positively charged Arg and from negatively-charged Glu to neutral Gly.


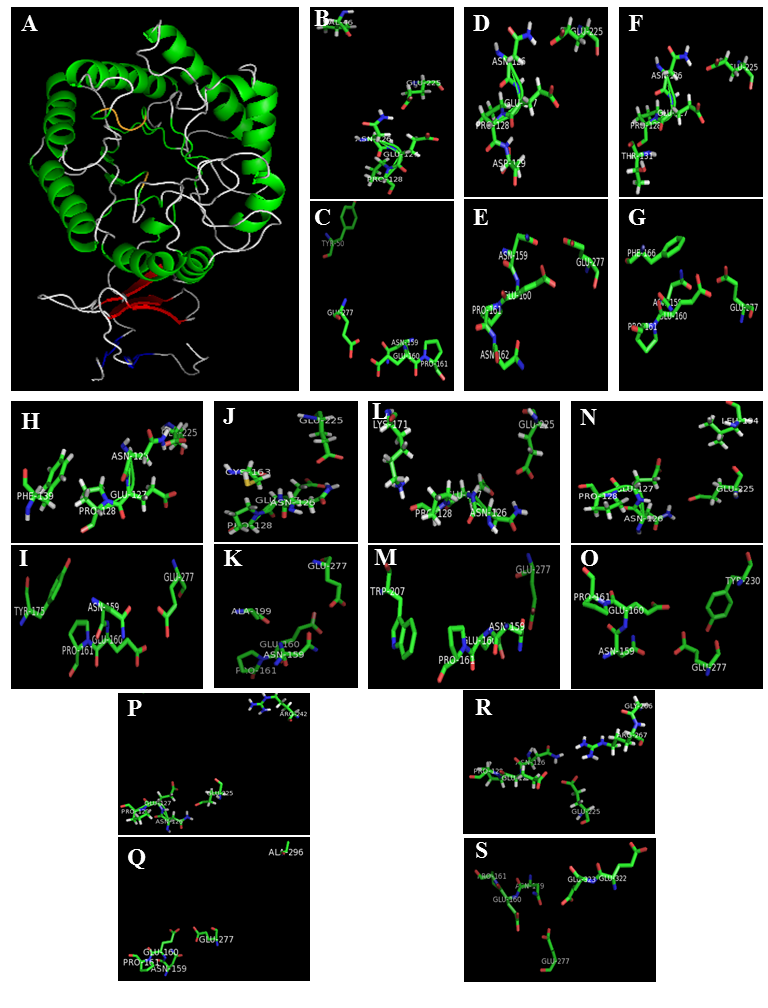


**
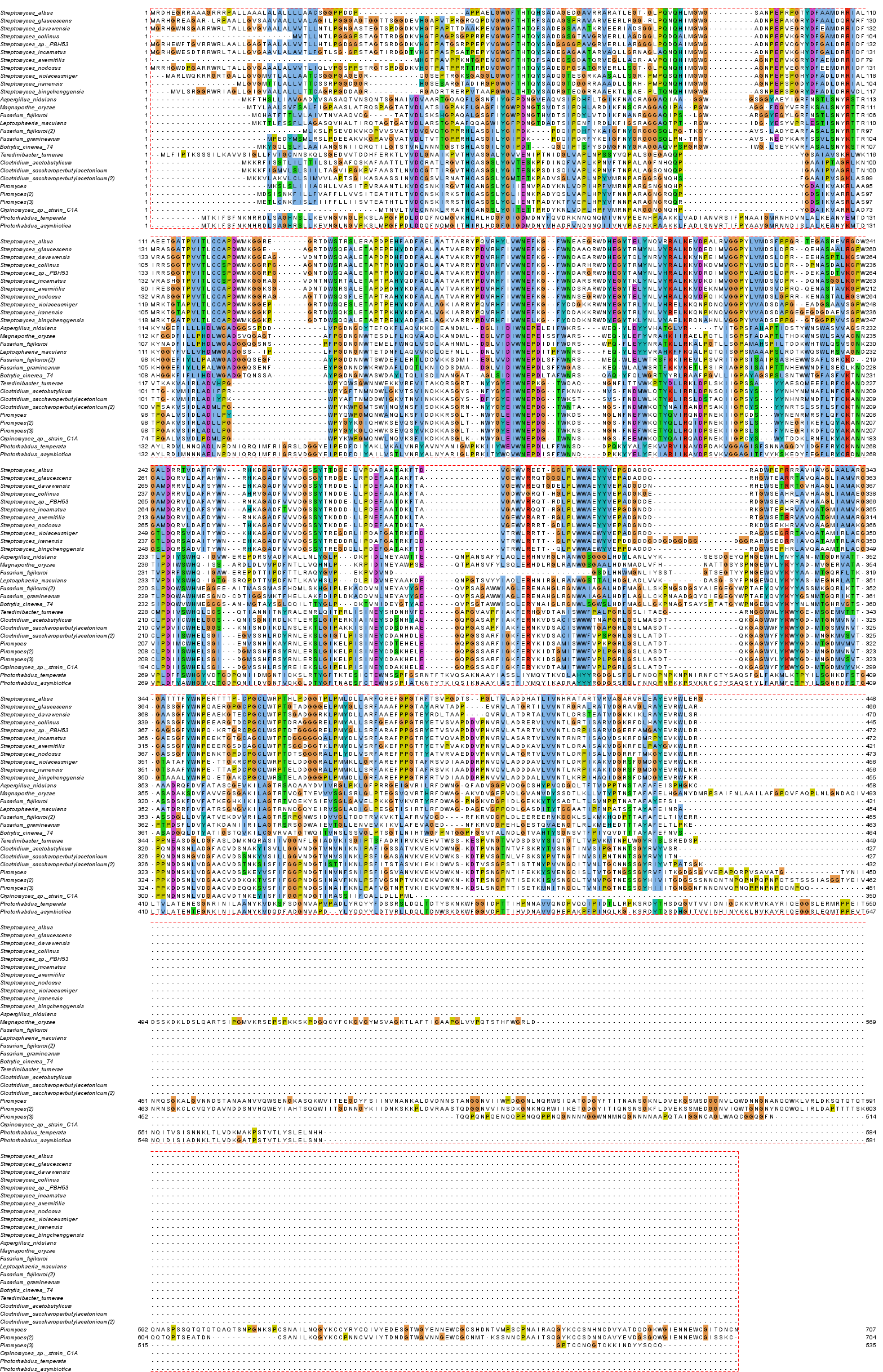
**

**Figure S5. Alignment of Class III enzymes (see Fig. 1).**

**Supplemental Material References**

Ahmed S, Bashir A, Saleem H, Saadia M, and Jamil A. 2009. Production and purification of cellulose-degrading enzymes from a filamentous fungus *Trichoderma harzianum*. *Pak J Bot* 41:1411-1419.

Ait N, Creuzet N, and Cattaneo J. 1982. Properties of beta-glucosidase purified from *Clostridium thermocellum*. *J Gen Microbiol* 128:569-577.

Amouri B, and Gargouri A. 2006. Characterization of a novel beta-glucosidase from a *Stachybotrys* strain. *Biochem Eng J* 32:191-197. 10.1016/j.bej.2006.09.022

Archana A, and Satyanarayana T. 2003. Purification and characterization of a cellulase-free xylanase of a moderate thermophile *Bacillus licheniformis* A99. *World J Microb Biot* 19:53-57. Doi 10.1023/A:1022527702400

Ariffin H, Abdullah N, Umi Kalsom MS, Shirai Y, and Hassan MA. 2006. Production and characterization of cellulase by *Bacillus pumilus* EB3. *Int J Res Eng Technol* 3:47-53.

Bagga PS, Sandhu DK, and Sharma S. 1990. Purification and characterization of cellulolytic enzymes produced by *Aspergillus nidulans*. *J Appl Bacteriol* 68:61-68. DOI 10.1111/j.1365-2672.1990.tb02549.x

Bailey MJ, Siikaaho M, Valkeajarvi A, and Penttila ME. 1993. Hydrolytic properties of 2 cellulases of *Trichoderma reesei* expressed in yeast. *Biotechnol Appl Bioc* 17:65-76.

Baraznenok VA, Becker EG, Ankudimova NV, and Okunev NN. 1999. Characterization of neutral xylanases from *Chaetomium cellulolyticum* and their biobleaching effect on eucalyptus pulp. *Enzyme Microb Technol* 25:651-659. Doi 10.1016/S0141-0229(99)00091-5

Bhalla A, Bischoff KM, and Sani RK. 2014. Highly thermostable GH39 beta-xylosidase from a *Geobacillus* sp strain WSUCF1. *BMC Biotechnol* 14:963-973. Artn 963 10.1186/S12896-014-0106-8

Bhat KM, Gaikwad JS, and Maheshwari R. 1993. Purification and characterization of an extracellular beta-glucosidase from the thermophilic fungus *Sporotrichum thermophile* and its influence on cellulase activity. *J Gen Microbiol* 139:2825-2832.

Bok JD, Yernool DA, and Eveleigh DE. 1998. Purification, characterization, and molecular analysis of thermostable cellulases CelA and CelB from *Thermotoga neapolitana*. *Appl Environ Microbiol* 64:4774-4781.

Bronnenmeier K, Rucknagel KP, and Staudenbauer WL. 1991. Purification and properties of a novel type of exo-1,4-beta-glucanase (avicelase II) from the cellulolytic thermophile *Clostridium stercorarium*. *Eur J Biochem* 200:379-385.

Bronnenmeier K, and Staudenbauer WL. 1988. Purification and properties of an extracellular beta-glucosidase from the cellulolytic thermophile *Clostridium stercorarium*. *Appl Microbiol Biot* 28:380-386.

Bukhtojarov FE, Ustinov BB, Salanovich TN, Antonov AI, Gusakov AV, Okunev ON, and Sinitsyn AP. 2004. Cellulase complex of the fungus *Chrysosporium lucknowense*: Isolation and characterization of endoglucanases and cellobiohydrolases. *Biochemistry* 69:542-551. Doi 10.1023/B:Biry.0000029853.34093.13

Cardoso OAV, and Ferreira EX. 2003. Purification and characterization of a novel cellulase-free xylanase from *Acrophialophora nainiana*. *FEMS Microbiol Lett* 223:309-314. 10.1016/S0378-1097(03)00392-6

Chen HZ, Li XL, and Ljungdahl LG. 1994. Isolation and properties of an extracellular beta-glucosidase from the polycentric rumen fungus *Orpinomyces s*p strain pc-2. *Appl Environ Microbiol* 60:64-70.

Christakopoulos P, Kekos D, Macris BJ, Claeyssens M, and Bhat MK. 1996. Purification and characterisation of a major xylanase with cellulase and transferase activities from *Fusarium oxysporum*. *Carbohyd Res* 289:91-104. Doi 10.1016/0008-6215(96)00146-2

Citti JE, Sandine WE, and Elliker PR. 1965. Beta-galactosidase of *Streptococcus lactis*. *J Bacteriol* 89:937-942.

Cohen R, Suzuki MR, and Hammel KE. 2005. Processive endoglucanase active in crystalline cellulose hydrolysis by the brown rot basidiomycete *Gloeophyllum trabeum*. *Appl Environ Microbiol* 71:2412-2417. 10.1128/Aem.71.5.2412-2417.2005

Correa JM, Graciano L, Abrahao J, Loth EA, Gandra RF, Kadowaki MK, Henn C, and Simao RDG. 2012. Expression and characterization of a GH39 beta-xylosidase II from *Caulobacter crescentus*. *Appl Biochem Biotech* 168:2218-2229. 10.1007/s12010-012-9931-1

Curry C, Gilkes N, O'Neill G, Miller RC, and Skipper N. 1988. Expression and secretion of a *Cellulomonas fimi* exoglucanase in *Saccharomyces cerevisiae*. *Appl Environ Microbiol* 54:476-484.

Czjzek M, Ben David A, Braman T, Shoham G, Henrissat B, and Shoham Y. 2005. Enzyme-substrate complex structures of a GH39 beta-xylosidase from *Geobacillus stearothermophilus*. *J Mol Biol* 353:838-846. 10.1016/j.jmb.2005.09.003

Deshpande V, Lachke A, Mishra C, Keskar S, and Rao M. 1986. Mode of action and properties of xylanase and beta-xylosidase from *Neurospora crassa*. *Biotechnol Bioeng* 28:1832-1837. DOI 10.1002/bit.260281210

Dhillon A, Gupta JK, and Khanna S. 2000. Enhanced production, purification and characterisation of a novel cellulase-poor thermostable, alkalitolerant xylanase from *Bacillus circulans* AB 16. *Process Biochem* 35:849-856. Doi 10.1016/S0032-9592(99)00152-1

Dobberstein J, and Emeis CC. 1991. Purification and characterization of beta-xylosidase from *Aureobasidium pullulans*. *Appl Microbiol Biot* 35:210-215.

Dutta T, Sengupta R, Sahoo R, Ray SS, Bhattacharjee A, and Ghosh S. 2007. A novel cellulase free alkaliphilic xylanase from alkali tolerant *Penicillium citrinum*: production, purification and characterization. *Lett Appl Microbiol* 44:206-211. 10.1111/j.1472-765X.2006.02042.x

Gamauf C, Marchetti M, Kallio J, Puranen T, Vehmaanpera J, Allmaier G, Kubicek CP, and Seiboth B. 2007. Characterization of the bga1-encoded glycoside hydrolase family 35 beta-galactosidase of *Hypocrea jecorina* with galacto-beta-D-galactanase activity. *FEBS J* 274:1691-1700. 10.1111/j.1742-4658.2007.05714.x

Garcia-Campayo V, and Wood TM. 1993. Purification and characterization of a beta-D-xylosidase from the anaerobic rumen fungus *Neocallimastix frontalis*. *Carbohyd Res* 242:229-245. Doi 10.1016/0008-6215(93)80037-F

Gardner RM, Doerner KC, and White BA. 1987. Purification and characterization of an exo-beta-1,4-glucanase from *Ruminococcus flavefaciens* FD-1. *J Bacteriol* 169:4581-4588.

Hakamada Y, Koike K, Yoshimatsu T, Mori H, Kobayashi T, and Ito S. 1997. Thermostable alkaline cellulase from an alkaliphilic isolate, *Bacillus* sp. KSM-S237. *Extremophiles* 1:151-156. DOI 10.1007/s007920050028

Harhangi HR, Steenbakkers PJM, Akhmanova A, Jetten MSM, van der Drift C, and Op den Camp HJM. 2002. A highly expressed family 1 beta-glucosidase with transglycosylation capacity from the anaerobic fungus *Piromyces* sp E2. *BBA-Gene Struct Expr* 1574:293-303. Pii S0167-4781(01)00380-3

Doi 10.1016/S0167-4781(01)00380-3

Hayashi S, Ohno T, Ito M, and Yokoi H. 2001. Purification and properties of the cell-associated ss-xylosidase from *Aureobasidium*. *J Ind Microbiol Biot* 26:276-279. DOI 10.1038/sj.jim.7000120

Hebraud M, and Fevre M. 1988a. Characterization of Glycoside and Polysaccharide Hydrolases Secreted by the Rumen Anaerobic Fungi Neocallimastix-Frontalis, Sphaeromonas-Communis and Piromonas-Communis. *Journal of General Microbiology* 134:1123-1129.

Hebraud M, and Fevre M. 1988b. Characterization of glycoside and polysaccharide hydrolases secreted by the rumen anaerobic fungi *Neocallimastix frontalis*, *Sphaeromonas communis* and *Piromonas communis*. *J Gen Microbiol* 134:1123-1129.

Hebraud M, and Fevre M. 1990. Purification and characterization of an extracellular beta-xylosidase from the rumen anaerobic fungus *Neocallimastix frontalis*. *FEMS Microbiol Lett* 72:11-16. DOI 10.1111/j.1574-6968.1990.tb03853.x

Hinz SWA, van den Broek LAM, Beldman G, Vincken JP, and Voragen AGJ. 2004. Beta-galactosidase from *Bifidobacterium adolescentis* DSM20083 prefers beta(1,4)-galactosides over lactose. *Appl Microbiol Biot* 66:276-284. 10.1007/s00253-004-1745-9

Huang XP, and Monk C. 2004. Purification and characterization of a cellulase (CMCase) from a newly isolated thermophilic aerobic bacterium *Caldibacillus cellulovorans* gen. nov., sp nov. *World J Microb Biot* 20:85-92. Doi 10.1023/B:Wibi.0000013316.12730.E7

Iembo T, Azevedo MO, Bloch C, and Filho EXF. 2006. Purification and partial characterization omicron f a new beta-xylosidase from *Humicola grisea var. thermoidea*. *World J Microb Biot* 22:475-479. 10.1007/s11274-005-9059-3

John M, Schmidt B, and Schmidt J. 1979. Purification and some properties of five endo-1,4-beta-D-xylanases and a beta-D-xylosidase produced by a strain of *Aspergillus niger*. *Can J Biochem Cell B* 57:125-134.

Jorgensen H, Eriksson T, Borjesson J, Tjerneld F, and Olsson L. 2003. Purification and characterization of five cellulases and one xylanase from *Penicillium brasilianum* IBT 20888. *Enzyme Microb Tech* 32:851-861. 10.1016/S0141-0229(03)00056-5

Kiss T, and Kiss L. 2000. Purification and characterization of an extracellular beta-D-xylosidase from *Aspergillus carbonarius*. *World J Microb Biot* 16:465-470. Doi 10.1023/A:1008912025896

Kitpreechavanich V, Hayashi M, and Nagai S. 1986. Purification and characterization of extracellular beta-xylosidase and beta-glucosidase from *Aspergillus fumigatus*. *Agr Biol Chem* 50:1703-1711.

Kormelink FJM, Searlevanleeuwen MJE, Wood TM, and Voragen AGJ. 1993. Purification and characterization of 3 endo-(1,4)-beta-xylanases and one beta-xylosidase from *Aspergillus awamori*. *J Biotechnol* 27:249-265. Doi 10.1016/0168-1656(93)90089-6

Kumar S, and Ramon D. 1996. Purification and regulation of the synthesis of a beta-xylosidase from *Aspergillus nidulans*. *FEMS Microbiol Lett* 135:287-293. DOI 10.1111/j.1574-6968.1996.tb08003.x

Ladero M, Santos A, Garcia JL, Carrascosa AV, Pessela BCC, and Garcia-Ochoa F. 2002. Studies on the activity and the stability of beta-galactosidases from *Thermus* sp strain T2 and from *Kluyveromyces fragilis*. *Enzyme Microb Tech* 30:392-405. Pii S0141-0229(01)00506-3

Doi 10.1016/S0141-0229(01)00506-3

Lama L, Calandrelli V, Gambacorta A, and Nicolaus B. 2004. Purification and characterization of thermostable xylanase and beta-xylosidase by the thermophilic bacterium *Bacillus thermantarcticus*. *Res Microbiol* 155:283-289. 10.1016/j.resmic.2004.02.001

Lee RC, Hrmova M, Burton RA, Lahnstein J, and Fincher GB. 2003. Bifunctional family 3 glycoside hydrolases from barley with alpha-L-arabinofuranosidase and beta-D-xylosidase activity - characterization, primary structures, and COOH-terminal processing. *J Biol Chem* 278:5377-5387. 10.1074/jbc.M210627200

Lee YJ, Kim BK, Lee BH, Jo KI, Lee NK, Chung CH, Lee YC, and Lee JW. 2008. Purification and characterization of cellulase produced by *Bacillus amyoliquefaciens* DL-3 utilizing rice hull. *Bioresource Technol* 99:378-386. 10.1016/j.biortech.2006.12.013

Li LT, Tian HM, Cheng YQ, Jiang ZQ, and Yang SQ. 2006. Purification and characterization of a thermostable cellulase-free xylanase from the newly isolated *Paecilomyces themophila*. *Enzyme Microb Tech* 38:780-787. 10.1016/j.enzmictec.2005.08.007

Li XT, She YL, Sun BG, Song HL, Zhu YP, Lv YG, and Song HX. 2010. Purification and characterization of a cellulase-free, thermostable xylanase from *Streptomyces rameus* L2001 and its biobleaching effect on wheat straw pulp. *Biochem Eng J* 52:71-78. 10.1016/j.bej.2010.07.006

Liebl W, Ruile P, Bronnenmeier K, Riedel K, Lottspeich F, and Greif I. 1996. Analysis of a *Thermotoga maritima* DNA fragment encoding two similar thermostable cellulases, CelA and CelB, and characterization of the recombinant enzymes. *Microbiol-SGM* 142:2533-2542.

Lucena-Neto SD, and Ferreira EX. 2004. Purification and characterization of a new xylanase from *Humicola grisea var. Thermoidea*. *Braz J Microbiol* 35:86-90. Doi 10.1590/S1517-83822004000100014

Lymar ES, Li B, and Renganathan V. 1995. Purification and characterization of a cellulose-binding beta-glucosidase from cellulose-degrading cultures of *Phanerochaete chrysosporium*. *Appl Environ Microbiol* 61:2976-2980.

Macgregor RB, and Weber G. 1986. Estimation of the Polarity of the Protein Interior by Optical Spectroscopy. *Nature* 319:70-73. Doi 10.1038/319070a0

Manzanares P, de Graaff LH, and Visser J. 1998. Characterization of galactosidases from *Aspergillus niger*: purification of a novel alpha-galactosidase activity. *Enzyme Microb Tech* 22:383-390. Doi 10.1016/S0141-0229(97)00207-X

Matsuo M, Fujie A, Win M, and Yasui T. 1987. 4 types of beta-xylosidases from *Penicillium wortmanni* ifo-7237. *Agr Biol Chem* 51:2367-2379.

Matsuo M, and Yasui T. 1984. Purification and some properties of beta-xylosidase from *Trichoderma viride*. *Agr Biol Chem* 48:1845-1852.

Meyer HP, and Canevascini G. 1981. Separation and some properties of 2 intracellular beta-glucosidases of *Sporotrichum* (*Chrysosporium*) thermophile. *Appl Environ Microbiol* 41:924-931.

Monti R, Terenzi HF, and Jorge JA. 1991. Purification and properties of an extracellular xylanase from the thermophilic fungus *Humicola grisea var thermoidea*. *Can J Microbiol* 37:675-681.

Morosoli R, Bertrand JL, Mondou F, Shareck F, and Kluepfel D. 1986. Purification and properties of a xylanase from *Streptomyces lividans*. *Biochem J* 239:587-592.

Nanmori T, Watanabe T, Shinke R, Kohno A, and Kawamura Y. 1990. Purification and properties of thermostable xylanase and beta-xylosidase produced by a newly isolated *Bacillus stearothermophilus* strain. *J Bacteriol* 172:6669-6672.

Onishi N, and Tanaka T. 1995. Purification and properties of a novel thermostable galacto-oligosaccharide-producing beta-galactosidase from *Sterigmatomyces elviae* cbs8119. *Appl Environ Microbiol* 61:4026-4030.

Parry NJ, Beever DE, Owen E, Vandenberghe I, Van Beeumen J, and Bhat MK. 2001. Biochemical characterization and mechanism of action of a thermostable beta-glucosidase purified from *Thermoascus aurantiacus*. *Biochem J* 353:117-127.

Pitson SM, Seviour RJ, and McDougall BM. 1997. Purification and characterization of an extracellular beta-glucosidase from the filamentous fungus *Acremonium persicinum* and its probable role in beta-glucan degradation. *Enzyme Microb Tech* 21:182-190. Doi 10.1016/S0141-0229(96)00263-3

Poutanen K, and Puls J. 1988. Characteristics of *Trichoderma reesei* beta-xylosidase and its use in the hydrolysis of solubilized xylans. *Appl Microbiol Biot* 28:425-432.

Rizzatti ACS, Jorge JA, Terenzi HF, Rechia CGV, and Polizeli MLTM. 2001. Purification and properties of a thermostable extracellular beta-D-xylosidase produced by a thermotolerant Aspergillus phoenicis. *Journal of Industrial Microbiology & Biotechnology* 26:156-160. DOI 10.1038/sj.jim.7000107

Saha BC. 2001. Purification and characterization of an extracellular beta-xylosidase from a newly isolated *Fusarium verticillioides*. *J Ind Microbiol Biot* 27:241-245. DOI 10.1038/sj.jim.7000184

Saha BC. 2003. Purification and properties of an extracellular beta-xylosidase from a newly isolated *Fusarium proliferatum*. *Bioresource Technol* 90:33-38. 10.1016/S0960-8524(03)00098-1

Saha BC, and Bothast RJ. 1996. Production, purification, and characterization of a highly glucose-tolerant novel beta-glucosidase from *Candida peltata*. *Appl Environ Microbiol* 62:3165-3170.

Sakka K, Yoshikawa K, Kojima Y, Karita S, Ohmiya K, and Shimada K. 1993. Nucleotide sequence of the *Clostridium stercorarium* xyla gene encoding a bifunctional protein with beta-D-xylosidase and alpha-L-arabinofuranosidase activities, and properties of the translated product. *Biosci Biotech Bioch* 57:268-272.

Salles BC, Cunha RB, Fontes W, Sousa MV, and Filho EXF. 2000. Purification and characterization of a new xylanase from *Acrophialophora nainiana*. *J Biotechnol* 81:199-204. Doi 10.1016/S0168-1656(00)00280-7

Scrivener AM, and Slaytor M. 1994. Properties of the endogenous cellulase from *Panesthia cribrata saussure* and purification of major endo-beta-1,4-glucanase components. *Insect Biochem Molec* 24:223-231. Doi 10.1016/0965-1748(94)90001-9

Shao WL, and Wiegel J. 1992. Purification and characterization of a thermostable beta-xylosidase from *Thermoanaerobacter ethanolicus*. *J Bacteriol* 174:5848-5853.

Shao WL, Xue YM, Wu AL, Kataeva I, Pei JJ, Wu HW, and Wiegel J. 2011. Characterization of a bovel beta-xylosidase, XylC, from *Thermoanaerobacterium saccharolyticum* JW/SL-YS485. *Appl Environ Microbiol* 77:719-726. 10.1128/Aem.01511-10

Silveira FQD, de Sousa MV, Ricart CAO, Milagres AMF, de Medeiros CL, and Filho EXF. 1999. A new xylanase from a *Trichoderma harzianum* strain. *J Ind Microbiol Biot* 23:682-685.

Singh J, Batra N, and Sobti RC. 2004. Purification and characterisation of alkaline cellulase produced by a novel isolate, *Bacillus sphaericus* JS1. *J Ind Microbiol Biot* 31:51-56. 10.1007/s10295-004-0114-0

Sulistyo J, Kamiyama Y, and Yasui T. 1995. Purification and some properties of *Aspergillus pulverulentus* beta-xylosidase with transxylosylation capacity. *J Ferment Bioeng* 79:17-22. Doi 10.1016/0922-338x(95)92737-W

Suurnakki A, Tenkanen M, Siika-Aho M, Niku-Paavola ML, Viikari L, and Buchert J. 2000. *Trichoderma reesei* cellulases and their core domains in the hydrolysis and modification of chemical pulp. *Cellulose* 7:189-209. Doi 10.1023/A:1009280109519

Takashima S, Iikura H, Nakamura A, Hidaka M, Masaki H, and Uozumi T. 1998. Overproduction of recombinant *Trichoderma reesei* cellulases by *Aspergillus oryzae* and their enzymatic properties. *J Biotechnol* 65:163-171. Doi 10.1016/S0168-1656(98)00084-4

Takashima S, Nakamura A, Hidaka M, Masaki H, and Uozumi T. 1999. Molecular cloning and expression of the novel fungal beta-glucosidase genes from *Humicola grisea* and *Trichoderma reesei*. *J Biochem* 125:728-736.

Takashima S, Nakamura A, Masaki H, and Uozumi T. 1996. Purification and characterization of cellulases from *Humicola grisea*. *Biosci Biotech Bioch* 60:77-82.

Tan LUL, Yu EKC, Louisseize GW, and Saddler JN. 1987. Inexpensive, rapid procedure for bulk purification of cellulase-free beta-1,4-D-xylanase of high specific activity. *Biotechnol Bioeng* 30:96-100. DOI 10.1002/bit.260300114

Tomme P, Kwan E, Gilkes NR, Kilburn DG, and Warren RA. 1996. Characterization of CenC, an enzyme from *Cellulomonas fimi* with both endo- and exoglucanase activities. *J Bacteriol* 178:4216-4223.

Tong CC, Cole AL, and Shepherd MG. 1980. Purification and properties of the cellulases from the thermophilic fungus *Thermoascus aurantiacus*. *Biochem J* 191:83-94.

Tsujibo H, Miyamoto K, Kuda T, Minami K, Sakamoto T, Hasegawa T, and Inamori Y. 1992. Purification, properties, and partial amino-acid-sequences of thermostable xylanases from *Streptomyces thermoviolaceus* opc-520. *Appl Environ Microbiol* 58:371-375.

Tuohy MG, Puls J, Claeyssens M, Vrsanska M, and Coughlan MP. 1993. The xylan-degrading enzyme-system of *Talaromyces emersonii* - novel enzymes with activity against aryl beta-D-xylosides and unsubstituted xylans. *Biochem J* 290:515-523.

Ulrich JT, Temple KL, and Mcfeters GA. 1972. Induction and characterization of beta-galactosidase in an extreme thermophile. *J Bacteriol* 110:691-698.

Utt EA, Eddy CK, Keshav KF, and Ingram LO. 1991. Sequencing and expression of the *Butyrivibrio fibrisolvens* xylb gene encoding a novel bifunctional protein with beta-D-xylosidase and alpha-L-arabinofuranosidase activities. *Appl Environ Microbiol* 57:1227-1234.

van Casteren WHM, Eimermann M, van den Broek LAM, Vincken JP, Schols HA, and Voragen AGJ. 2000. Purification and characterisation of a beta-galactosidase from *Aspergillus aculeatus* with activity towards (modified) exopolysaccharides from *Lactococcus lactis* subsp cremoris B39 and B891. *Carbohyd Res* 329:75-85. Doi 10.1016/S0008-6215(00)00152-X

vanPeij NNME, Brinkmann J, Vrsanska M, Visser J, and deGraaff LH. 1997. Beta-xylosidase activity, encoded by xlnD, is essential for complete hydrolysis of xylan by *Aspergillus niger* but not for induction of the xylanolytic enzyme spectrum. *Eur J Biochem* 245:164-173.

Wagschal K, Franqui-Espiet D, Lee CC, Robertson GH, and Wong DWS. 2008. Cloning, expression and characterization of a glycoside hydrolase family 39 xylosidase from Bacillus halodurans C-125. *Applied Biochemistry and Biotechnology* 146:69-78. 10.1007/s12010-007-8055-5

Wilson CA, Mccrae SI, and Wood TM. 1994. Characterization of a beta-D-glucosidase from the anaerobic rumen fungus *Neocallimastix frontalis* with particular reference to attack on cello-oligosaccharides. *J Biotechnol* 37:217-227. Doi 10.1016/0168-1656(94)90129-5

Wood TM. 1971. Cellulase of *Fusarium solani*. *Biochem J* 121:353-362.

Wood TM, and Mccrae SI. 1982a. Purification and some properties of a (1-]4)-beta-D-glucan glucohydrolase associated with the cellulase from the fungus *Penicillium funiculosum*. *Carbohyd Res* 110:291-303. Doi 10.1016/0008-6215(82)84011-1

Wood TM, and Mccrae SI. 1982b. Purification and some properties of the extracellular beta-D-glucosidase of the celluloytic fungus *Trichoderma koningii*. *J Gen Microbiol* 128:2973-2982.

Woodward J, Brown JP, Evans BR, and Affholter KA. 1994. Papain digestion of crude *Trichoderma reesei* cellulase - purification and properties of cellobiohydrolase-I and cellobiohydrolase-II core proteins. *Biotechnol Appl Bioc* 19:141-153.

Ximenes FD, de Sousa MV, Puls J, da Silva FG, and Ferreira EX. 1999. Purification and characterization of a low-molecular-weight xylanase produced by *Acrophialophora nainiana*. *Curr Microbiol* 38:18-21. Doi 10.1007/Pl00006765

Ximenes FD, Silveira FQD, and Ximenes E. 1996. Production of beta-xylosidase activity by *Trichoderma harzianum* strains. *Curr Microbiol* 33:71-77. DOI 10.1007/s002849900077

Xu Z, Shih MC, and Poulton JE. 2006. An extracellular exo-beta-(1,3)-glucanase from *Pichia pastoris:* purification, characterization, molecular cloning, and functional expression. *Protein Expr Purif* 47:118-127. 10.1016/j.pep.2005.11.025

Yang S, Jiang Z, Yan Q, and Zhu H. 2008. Characterization of a thermostable extracellular beta-glucosidase with activities of exoglucanase and transglycosylation from *Paecilomyces thermophila*. *J Agric Food Chem* 56:602-608. 10.1021/jf072279+

Ye XY, Ng TB, and Cheng KJ. 2001. Purification and characterization of a cellulase from the ruminal fungus *Orpinomyces joyonii* cloned in *Escherichia coli*. *Int J Biochem Cell B* 33:87-94. Doi 10.1016/S1357-2725(00)00068-6

Yin LJ, Lin HH, and Xiao ZR. 2010. Purification and characterization of a cellulase from *Bacillus subtilis* yj1. *J Mar Sci Tech* 18:466-471.

Yuan TZ, Yang PL, Wang YR, Meng K, Luo HY, Zhang W, Wu NF, Fan YL, and Yao B. 2008. Heterologous expression of a gene encoding a thermostable beta-galactosidase from *Alicyclobacillus acidocaldarius*. *Biotechnol Lett* 30:343-348. 10.1007/s10529-007-9551-y

Zhou J, Wang YH, Chu J, Zhuang YP, Zhang SL, and Yin P. 2008. Identification and purification of the main components of cellulases from a mutant strain of *Trichoderma viride* T 100-14. *Bioresource Technol* 99:6826-6833. 10.1016/j.biortech.2008.01.077

Zhu H, Cheng KJ, and Forsberg CW. 1994. A truncated beta-xylosidase from the anaerobifungus *Neocallimastix patriciarum* 27. *Can J Microbiol* 40:484-490.

Zverlov V, Mahr S, Riedel K, and Bronnenmeier K. 1998. Properties and gene structure of a bifunctional cellulolytic enzyme (CelA) from the extreme thermophile '*Anaerocellum thermophilum*' with separate glycosyl hydrolase family 9 and 48 catalytic domains. *Microbiol* 144:457-465.
