# Supplementary material for: A multifunctional GH39 glycoside hydrolase from the anaerobic gut fungus Orpinomyces sp. strain C1A
Source: PeerJ. 2016 Aug 3;4:e2289. doi: 10.7717/peerj.2289 (PMC4975031; doi:10.7717/peerj.2289)
Supplement: Data S1 [file peerj-04-2289-s002.docx]

**Codon optimized sequence for Bgxg1 cloning in E coli:** ACCAACGTCCTGACGGTGGAATGCAACAATAAACTGCGTCGCGCGACCCATTGTGCCAATGGCAGCCTGTATGGTATTACGGAAACCACGCCGCGTGATTACAAATCTCTGGTTGACCCGCTGCATCCGTTTGTTATGCGTAATCCGGCCCGCGGCGGTAATGGTAATCAGCACCCGTATGGTGATGCAATCAAAGTGGCTCGTCGCCTGGCCGACACCCCGGGTGCACTGGTGAGTGTTGATCTGCCGGACATGCTGCCGGGTTGGCCGTATCGTTGGCCGGGTATGCAGAGTTGGCTGAACCAAGTCAAATCCTTCATCAACGATAAGAAAGCGTCCGGCCTGAAAAACTGGTATGGTCTGGAAATCTGGAATGAACCGGACGGCACCTGGAACAATTCAAATGGTTCGTTCGAAGAAATGTGGAAACAGACGTACCAAGTGATTCGTCAGGCCGATCCGAACGAAAAAATTATCGGTCCGTGCTATAGTTGGTACACCGATGACAAACTGCGCAATTTCCTGAAATACGCAAAAGCTAACAACTGTCTGCCGGATATTATCAGCTGGCATGAACTGTCTGGCATCGACGGTGTTAGCTCTCACCTGCGTTCATATCGCGAAATTGAAAAATCGCTGGGCATCCCGGAACTGCCGATTAGCATCAACGAATACTGCGATGCTGAACACGAACTGGAAGGCCAGCCGGGTAGTTCCGCGCGTTTCATCGGTAAATTCGAACGCTACAAAGTGGATACCGCGATGATCACGTGGTGGTTTGTTCCGTATCCGGGCCGTCTGGGTTCTCTGCTGGCAACCGATACGCAAAAAGGCGCAGGTTGGTATTTCTACAAATGGTATGGCGATATGACCGGTGACATGCTGTACGTCAAACCGCCGAACGATAATAGCAAACTGGTGGACGGCGCGGCCTGTCTGTAA
